# The longitudinal relationship between changes in wellbeing and inflammatory markers: Are associations independent of depression?

**DOI:** 10.1016/j.bbi.2019.10.004

**Published:** 2020-01

**Authors:** Daisy Fancourt, Andrew Steptoe

**Affiliations:** Department of Behavioural Science and Health, University College London, United Kingdom

**Keywords:** Inflammation, Depression, Wellbeing, Hedonic, Eudemonic, CRP, Fibrinogen, WBC

## Abstract

**Objectives:**

There is a large literature linking inflammation with mental ill health, but a much smaller literature focusing on mental wellbeing. Specifically, it remains unclear whether mental wellbeing is longitudinally and independently associated with inflammation or only via associated changes in mental ill health.

**Methods:**

This study used data from 8780 adults aged 50+ in the English Longitudinal Study of Ageing. Hedonic wellbeing (both positive affect and life satisfaction) and eudemonic wellbeing (self-realisation and control-autonomy) were measured at data collection waves 2 (2004/05), 4 (2008/09) and 6 (20012/13), along with measures of C-reactive protein (CRP), fibrinogen and white blood cells (WBC). Fixed effects modelling was performed to identify the longitudinal relationship between wellbeing and inflammation, adjusting for time-varying mental ill health and other identified confounders.

**Results:**

Both measured aspects of hedonic wellbeing were associated with lower WBC count, independent of mental ill health. For life satisfaction, this relationship was explained by confounders, whilst for positive affect it persisted. Both measured aspects of eudemonic wellbeing were associated with lower CRP, fibrinogen and WBC, independent of mental ill health. For control-autonomy, this relationship was explained by confounders, whilst for self-realisation it persisted. Results were present in both men and women, although more strongly in men, and were robust to a range of sensitivity analyses.

**Conclusions:**

This study builds on the strong literature showing a relationship between mental ill health and inflammation by showing that there is also an apparently independent relationship between mental wellbeing, in particular eudemonic wellbeing, and inflammation that is unexplained by socio-economic or other time-constant factors and in some instances persists independent of time-varying confounders.

## Highlights

• Hedonic wellbeing is longitudinally associated with lower WBC count.
• Eudemonic wellbeing is longitudinally associated with lower WBC, fibrinogen & CRP.
• Associations are independent of depression and other aspects of mental ill-health.
• Constant factors e.g. sex, medical history, SES & genetics cannot explain results.
• Associations are partly explained by time-varying health-related factors.

## 1 Introduction

A key hypothesis of in wellbeing research is that psychological wellbeing is accompanied by optimal functioning of multiple physiological systems (Ryff and Singer, 1998). Over the past decade, a number of studies have focused specifically on neuro-immune responses to wellbeing, with a growing interest in inflammation. There is a large literature showing that positive affect is associated with lower levels of inflammation, including levels of C-reactive protein (CRP) and interleukin 6 (IL-6), (Brouwers et al., 2013, Ironson et al., 2018, Ong et al., 2018, Stellar et al., 2015, Steptoe et al., 2008). Additionally, a small number of studies have expanded considerations to other aspects of wellbeing, finding similar patterns for life satisfaction in relation to CRP, IL-6 and fibrinogen (Hamer and Chida, 2011, Steptoe et al., 2012, Uchino et al., 2018), and aspects of eudemonic wellbeing such as purpose in relation to CRP, fibrinogen, WBC and soluble IL-6 receptor (sIL-6r) (Ryff et al., 2004, Steptoe et al., 2012, Steptoe and Fancourt, 2019). However, a number of questions remain.

First, most of this research has come from cross-sectional studies, the majority of which have involved relatively small samples (<500 participants). Only a very small number of studies have explored the relationship between wellbeing and inflammation over time. These have shown some similar patterns, such as an association between higher levels of overall subjective wellbeing and lower levels of CRP 2 years later in older adults (Okely et al., 2017), higher levels of positive affect and lower CRP over a 12 month period amongst a small cohort of breast cancer survivors (Moreno et al., 2016), and lower levels of positive affect during a brief period of induced stress and increased IL-1β reactivity in a small laboratory study (Aschbacher et al., 2012). But whether patterns of *change* in wellbeing and inflammation are related over longer periods and in larger samples remains unexplored.

Second, there is already a large literature on inflammation and mental illness, in particular depression. This relationship appears to be bidirectional: patients with inflammatory diseases have higher rates of major depressive disorder, around a third of individuals with major depression show elevated peripheral inflammatory biomarkers, and patients treated with inflammatory cytokines are at increased risk of developing depression (Amodeo et al., 2017, Dahl et al., 2014, Dowlati et al., 2010, Raison and Miller, 2013). Mental illness and mental wellbeing are well established as two distinct but related constructs (Keyes, 2005), but whether they have distinct relationships with biological markers has remained unclear (Ryff et al., 2006). Many studies on inflammation and wellbeing have not taken account of mental illness (Ong et al., 2018, Stellar et al., 2015, Uchino et al., 2018). Several others have found results attenuated when adjusting for depression (Brouwers et al., 2013, Ironson et al., 2018, Ryff et al., 2004), which could suggest that wellbeing is only related to inflammation via associated changes in mental ill-health. Indeed, some genetic studies have supported this hypothesis, by suggesting that the same genetic variants are associated with the propensity to feel positive about life as the propensity to feel depressed (Okbay et al., 2016). Similarly, epigenetic studies have shown that depression and hedonic wellbeing (life satisfaction and happiness) are associated with methylation of the same promotors such as intercellular adhesion molecule 1 (ICAM-1) and Tissue Factor (F3) in leukocytes (Kim et al., 2016), both of which are involved in inflammatory processes. However, it has also been argued that wellbeing is independently associated with inflammation. A few studies have found associations between wellbeing and inflammation persist even when accounting for depression (Hamer and Chida, 2011, Steptoe et al., 2012, Steptoe et al., 2008, Steptoe and Fancourt, 2019). This may be partly due to specific pathways to inflammation for certain aspects of wellbeing (for example, positive affect has been explored in relation to buffering against psychological stress (Blevins et al., 2017)). It may also be partly due to genetic evidence contrasting that presented above, which suggests that depression and wellbeing have only some shared genetic influences and are also influenced in part by independent genetic factors (Bartels et al., 2010, Kendler et al., 2011, Plomin et al., 1992). However, the studies that have been carried out on wellbeing and inflammation adjusting for depression have, to date, been confined to cross-sectional samples.

Finally, although various studies have considered multiple different aspects of wellbeing, very few have simultaneously compared associations between inflammation and different aspects of wellbeing such as hedonic wellbeing (which focuses on experienced aspects of wellbeing such as pleasure and evaluative aspects of wellbeing such as life satisfaction) vs eudemonic wellbeing (which focuses on meaning, self-realisation and flourishing in life). These components are related, with estimates of the magnitude of overlap between hedonia and eudemonia suggesting around half to three-quarters of the variance is common (Kashdan et al., 2008). Yet it is important to consider the differential association of each with inflammation given there are different theoretical backgrounds to both constructs and given research suggesting they may involve different biological pathways. For example, neurobiological work has shown that while both hedonic and eudemonic wellbeing are associated with greater left than right superior frontal EEG activity, eudemonic wellbeing explains variance beyond that accounted for by positive affect (Urry et al., 2004). This higher left-sided activation has been linked to immune response, such as in studies that have shown an association with higher antibody response to influenza vaccination challenge (Rosenkranz et al., 2003). At a genetic level, wellbeing has been proposed to be genetically and environmentally multidimensional, with different factors indicating hedonic and eudemonic components (Franz et al., 2012). So it is possible that different aspects of wellbeing may have differential associations with inflammation. Therefore, this study explored the longitudinal association between changes in different aspects of hedonic and eudemonic wellbeing and changes in inflammatory markers in a large sample of 8780 adults over an 8-year follow-up period. Further, given previous research has found sex differences in the association between wellbeing and inflammation (Steptoe et al., 2012), we considered whether there could be a moderating role of sex.

## 2 Methods

### 2.1 Participants

Participants were drawn from the English Longitudinal Study of Ageing (ELSA): a nationally representative panel study of adults aged 50 and above living in England (Steptoe et al., 2013). Participants were originally recruited from households selected via multi-stage stratified probability sampling to participate in the Health Survey for England (HSE) in 1998–2001 (wave 0). Biennial data collection since has included blood samples taken during nurse visits in waves 2 (2004/05), 4 (2008/09) and 6 (2012/13). Consequently, this analysis involved 8780 core participants who had provided data in wave 2 and followed them up across waves 4 and 6, with multiple imputation used to maintain the same sample size across any missing data on the following two blood collection waves (see ‘Analysis’). The data are available through the UK Data Service and ethical approval for ELSA was provided by the National Research Ethics Service. All research was performed in accordance with research and data protection guidelines with all respondents providing informed consent.

### 2.2 Measures

#### 2.2.1 Wellbeing

Hedonic wellbeing was measured separately for positive affect and life satisfaction. Positive affect was measured using the pleasure sub-component of CASP-15; a validated scale that considers positive emotions and experiences in life (Vanhoutte, 2012). This includes items such as “I enjoy the things that I do” and “On balance I look back on my life with a sense of happiness.” CASP-15 was selected above CASP-19 as recommended by a specific factor analysis of wellbeing measures included within the English Longitudinal Study of Aging (Vanhoutte, 2014). Life satisfaction was measured using the Diener Satisfaction with Life Scale (SWLS) (Diener et al., 1985), which measures idiosyncratic judgement of an individual’s life. This includes items such as “In most ways my life is close to my ideal” and “I am satisfied with my life”. Eudemonic wellbeing was measured separately for self-realisation and control-autonomy using the respective subscales of CASP-15. Self-realisation considers meaning, purpose and growth within life, and was assessed with items such as “I feel that life is full of opportunities” and “I feel full of energy these days”. Control-autonomy considers the degree to which an individual can make their own decisions and lead the life they want to lead, and was assessed with items such as “I feel that what happens to me is out of my control” and “I can do the things I want to do”. For all four measures of wellbeing, scales were comprised of 5 items (each scored 0–4 for CASP-15 and 1–7 for SWLS), with higher scores indicating higher wellbeing.

#### 2.2.2 Neuro-immune Biomarkers

Blood samples collected during ELSA nurse visits were analysed to give data on three neuro-immune markers: white blood cell count (WBC; analysed as continuous counts per 10^9^/L), C-reactive protein (CRP; mg/L), and fibrinogen (g/L). CRP was measured using the N Latex CRP high-sensitivity mono immunoassay on the Behring Nephelometer II analyser. Fibrinogen was measured using a modification of the Clauss thrombin clotting method on the Organon Teknika MDA 180 analyser. All blood samples were analysed at the Royal Victoria Infirmary laboratory in Newcastle upon Tyne, UK (for a detailed description of blood analyses see Graig et al., 2004). For CRP levels, we excluded data from participants with results of higher than 10 mg/L, since these may indicate the presence of an acute infection or serious acute illness, and all results were log-transformed to ensure a normal distribution. Other biomarkers showed a normal distribution. WBC levels were reported at waves 4 (2008/09) and 6 (2012/13) only, therefore multiple imputation was additionally used to provide values at wave 2 (2004/05). Sensitivity analyses testing the consistency of results when just using data from waves 4 and 6 found no material difference, so imputed results are presented here and results excluding wave 2 imputations are shown in supplementary material.

#### 2.2.3 Confounders

Our statistical approach automatically took account of all time-constant factors even if they were unobserved, including age, sex, ethnicity, education, socio-economic status, genetics, past history of mental illness, and past history of wellbeing. Additionally, our models adjusted for depression (using a score of 4+ on the Centre for Epidemiological Studies Depression scale CES-D, which has previously been validated against gold-standard psychiatric interviews with good sensitivity and specificity (Radloff, 1977)), previous physician diagnosis of any other psychiatric condition (including anxiety, psychosis, major depression, mood swings or other emotional problems), current physician diagnosis of a long standing illnesses (including cancer, COPD, arthritis, stroke, diabetes and angina), whether participants currently smoked (yes vs no), whether participants were sedentary in their behaviours (classed as engaging in mild, moderate or vigorous sports or activities less than once a week), and body mass index (BMI).

### 2.3 Analysis

A total of 37% of data items were missing and assumed to be missing at random so were imputed in long format using multiple imputation by chained equations using the following predictor variables: demographic factors (age, sex, ethnicity), socio-economic factors (wealth, employment status, educational status, occupational status), health (alcohol consumption, smoking status, sedentary behaviours, chronic conditions, chronic pain, depression, other psychiatric condition), wellbeing (life satisfaction, pleasure, self-realisation, control-autonomy), social factors (marital status, loneliness, isolation), and biomarkers (pulse, systolic blood pressure, diastolic blood pressure, waist circumference, BMI, waist-height ratio, triglycerides, HbA1c levels, cholesterol/HDL ratio, CRP, fibrinogen, WBC, IGF-1). We undertook a single set of imputations for all missing data together (including exposure and outcome variables according to recommendations (Moons et al., 2006, Sterne et al., 2009)) and 40 imputations were conducted.

The relationship between within-person changes in wellbeing and within-person changes in biomarkers was measured using fixed-effects (FE) regression. FE regression has several strengths, including (i) it considers time-varying relationships between exposures, covariates and outcomes, which is especially helpful for factors that are likely to change over time; (ii) it considers within-person variation, with each individual acting as their own reference point over time. Therefore all time-invariant factors (e.g. sex, ethnicity, genetics, personality, educational attainment and socio-economic position) are accounted for even if unobserved (Allison, 2009), so do not need to be included in statistical models (and are shown in Table 1 purely for descriptive purposes). The basic model for the analysis can be expressed as follows:

$${\mathrm{Bio}}_{\mathrm{it}}={\beta_{0t}+\beta_{1}W}_{\mathrm{it}}+\beta_{2}T_{\mathrm{it}}+\alpha_{i}+\varepsilon_{\mathrm{it}}$$

where Bio_it_ is a measure of individual i's levels of neuro-immune markers at time t, α_i_ is unobserved time invariant confounding factors, W is an individual’s levels of wellbeing at time t, and T is measured time-varying confounding factors. Data were strongly balanced. A Hausman test was used to confirm the selection of a fixed effects over a random effects model. The modified Wald test for group-wise heteroscedasticity was significant, so sandwich estimators were applied. Coefficients for all years were not jointly equal to zero, so time-fixed effects were included in the model. Analyses were carried out using Stata 14 (StataCorp, College Station, TX).

**Table 1:** Demographic characteristics of the sample.

		Proportion (%)/Mean (SE)
Time-constant factors*		
Age	50–54	12.5%
	55–59	21.3%
	60–64	16.1%
	65–69	15.9%
	70–74	13.7%
	75+	20.6%
Sex	% female	54.5%
Ethnicity	% White British	97.8%
Educational attainment	No qualifications/basic qualifications	43.7%
	GCSE/O-level/qualification at age 16	16.8%
	A-levels/higher education/qualification at age 18	27.3%
	Degree/further higher qualification	12.2%
Social status	Managerial, administrative and professional occupations	30.9%
	Intermediate occupations	14.0%
	Small employers and own account workers	10.8%
	Lower supervisory and technical occupations	11.0%
	Semi-routine and routine occupations	33.3%

Time-varying exposures and outcomes**		
Positive affect	Range 0–20 (higher indicates higher wellbeing)	13.3 (0.03)
Life satisfaction	Range 5–35 (higher indicates higher wellbeing)	25.9 (0.07)
Self-realisation	Range 0–20 (higher indicates higher wellbeing)	9.9 (0.04)
Control-autonomy	Range 0–20 (higher indicates higher wellbeing)	11.1 (0.03)
CRP	mg/L; log-transformed	1.14 (0.01)
Fibrinogen	g/L	3.24 (0.01)
WBC	counts per 10^9^/L	6.51 (0.03)

Time-varying confounders**		
Depression	% score of 4+ in CES-D	15.5%
Other psychiatric condition	% diagnosed with anxiety, psychosis, major depression, mood swings or other emotional problems	3.8%
Chronic Health Conditions	One or more of cancer, COPD, arthritis, stroke, diabetes, angina, %	36.9%
Sedentary Behaviour	Exercises less than weekly, %	9.7%
Smoking Status	% current smoker	15.1%
BMI		28.0 (0.06)

*Automatically included within the FE model, but shown for descriptive purposes. **Values shown at baseline. SE: standard error. CRP: C-Reactive Protein; WBC: white blood cell count.

Model 1 was adjusted only for time-constant factors, which were included automatically within the model. Model 2 additionally adjusted for depression and other psychiatric conditions. Model 3 additionally adjusted for chronic illness, smoking, sedentary behaviours, and BMI. Our first sensitivity analysis tested the consistency of findings when using a continuous scale of depressive symptoms rather than a binary score for depression. Our second sensitivity analysis tested whether our results for WBC count were just an artefact of our imputation of data for wave 2, so we re-ran analyses just focusing on waves 4 and 6. Our third sensitivity analysis excluded any participants who had an acute infection in the three weeks preceding blood sampling. Finally, we explored if there was any moderation by sex, age or BMI by including interaction terms within the models and, where significant, stratifying and re-running analyses.

## 3 Results

### 3.1 Sample

Our sample was aged 50 and above, 55% female, and 98% white British (Table 1). Amongst the sample, there was a large association between all aspects of wellbeing. Within hedonic wellbeing, pleasure and life satisfaction shared 31% variance (r = 0.55, p < .001), while within eudemonic wellbeing, self-realisation and autonomy shared 42% variance (r = 0.65, p < .001). Across the domains of wellbeing, pleasure shared 45% variance with self-realisation (r = 0.67, p < .001) and 30% with control-autonomy (r = 0.55, p < .001), and life satisfaction shared 41% variance with self-realisation (r = 0.64, p < .001) and 30% with control-autonomy (r = 0.54, p < .001).

### 3.2 Hedonic wellbeing & inflammation

For positive affect, changes over time were negatively associated with changes in WBC when adjusted for time-constant factors only (Table 2). When additionally for mental ill health, the association was maintained with only an 8% decrease in the coefficient. The association was also maintained when adjusting for time-varying covariates. However, there was no association between changes in positive affect and changes in either CRP or fibrinogen in any of the models.

**Table 2:** Fixed effects models showing associations between changes in wellbeing and changes in inflammatory markers.

	CRP			Fibrinogen			WBC		
	Coef	95% CI	p	Coef	95% CI	p	Coef	95% CI	p
*Adjusted for time-constant factors*									
Positive affect	−0.004	−0.0108,0.0029	0.25	−0.0059	−0.0127,0.0010	0.094	**−0.0558**	**−0.0799,-0.0317**	**<0.001**
Life satisfaction	−0.0012	−0.0037,0.0012	0.32	−0.0016	−0.0044,0.0012	0.26	**−0.0139**	**−0.0233,-0.0046**	**0.004**
Self-realisation	**−0.0142**	**−0.0190,-0.0094**	**<0.001**	**−0.0147**	**−0.0200,-0.0095**	**<0.001**	**−0.0648**	**−0.0835,-0.0461**	**<0.001**
Control-autonomy	**−0.0097**	**−0.0145,-0.0049**	**<0.001**	**−0.0121**	**−0.0179,-0.0063**	**<0.001**	**−0.0348**	**−0.0561,-0.0135**	**0.002**

*Additionally adjusted for mental ill-health*									
Positive affect	−0.0006	−0.0077,0.0066	0.87	−0.0024	−0.0092,0.0044	0.49	**−0.0511**	**−0.0766,-0.0256**	**<0.001**
Life satisfaction	0.0001	−0.0025,0.0026	0.95	−0.0002	−0.0031,0.0027	0.90	**−0.0115**	**−0.0214,-0.0016**	**0.023**
Self-realisation	**−0.0126**	**−0.0177,-0.0075**	**<0.001**	**−0.0128**	**−0.0182,-0.0074**	**<0.001**	**−0.0629**	**−0.0831,-0.0427**	**<0.001**
Control-autonomy	**−0.0076**	**−0.0127,-0.0025**	**0.004**	**−0.0099**	**−0.0156,-0.0042**	**0.001**	**−0.0301**	**−0.0532,-0.0069**	**0.012**

*Additionally adjusted for covariates*									
Positive affect	0.0023	−0.0048,0.0093	0.53	0.0013	−0.0054,0.0080	0.70	**−0.0342**	**−0.0590,-0.0093**	**0.008**
Life satisfaction	0.0019	−0.0006,0.0043	0.14	0.0015	−0.0014,0.0044	0.30	−0.0048	−0.0146,0.0049	0.33
Self-realisation	**−0.0074**	**−0.0125,-0.0024**	**0.004**	**−0.0076**	**−0.0130,-0.0022**	**0.006**	**−0.0439**	**−0.0640,-0.0238**	**<0.001**
Control-autonomy	−0.0032	−0.0085,0.0020	0.23	−0.0056	−0.0115,0.0002	0.06	−0.0142	−0.0369,0.0084	0.22

N = 8780. Observations = 26,340. CRP: C-Reactive Protein; WBC: white blood cell count; Unadjusted models automatically account for all factors that are time-constant in older age, including age, sex, ethnicity, education, socio-economic status, genetics, past history of mental illness, and past history of wellbeing. Mental ill health includes depression and other psychiatric conditions. Additional covariates include chronic conditions, BMI and smoking. Boldface indicates p < .05.

For life satisfaction, changes over time were negatively associated with changes in WBC when adjusted for time-constant factors only. When additionally for mental ill health, the association was maintained with only a 17% decrease in the coefficient. However, the association was explained by time-varying covariates. There was no association between changes in positive affect and changes in either CRP or fibrinogen in any of the models.

### 3.3 Eudemonic wellbeing & inflammation

For self-realisation, changes over time were negatively associated with changes in CRP, fibrinogen and WBC when adjusted for time-constant factors only (Table 2). When additionally for mental ill health, the association was maintained with only an 11% decrease in the coefficient for CRP, 13% for fibrinogen and 3% for WBC. The association with all three biomarkers was also maintained when adjusting for time-varying covariates.

For control-autonomy, changes over time were negatively associated with changes in CRP, fibrinogen and WBC when adjusted for time-constant factors only. When additionally for mental ill health, the association was maintained with only a 22% decrease in the coefficient for CRP, 18% for fibrinogen and 14% for WBC. However, the association with all three biomarkers was explained when adjusting for time-varying covariates.

### 3.4 Sensitivity analyses

Results were materially unchanged when using depression as a continuous variable rather than a binary variable (Supplementary Table 1). When excluding individuals who had experienced an acute infection in the three weeks prior to blood sampling, the associations for all aspects of wellbeing and all biomarkers were maintained when adjusting just for time-constant factors and when additionally adjusting for mental ill health, but there was some attenuation when additionally adjusting for covariates (Supplementary Table 2). For WBC, when re-running analyses just using waves 4 and 6 (as results were entirely imputed for wave 2 due to this biomarker not being analysed), results were maintained, with the exception of some attenuation when additionally adjusting for covariates (Supplementary Table 3).

There was evidence of moderation by sex, but stratified analyses showed that although there were slightly stronger results in men, they were still significantly present in women (Table 3). Similarly, there was evidence of moderation by age and BMI. Although there were slightly stronger in those aged over 65 and those who were overweight or obese, there was still a clear association between self-realisation and WBC in those aged under 65 and those with a healthy BMI (Supplementary Tables 4 & 5).

**Table 3:** Fixed effects models showing associations between changes in wellbeing and changes in inflammation: split by sex.

	CRP			Fibrinogen			WBC		
	Coef	95% CI	p	Coef	95% CI	p	Coef	95% CI	p
MEN (N = 3,494)									
*Adjusted for time-constant factors*									
Positive affect	−0.002	−0.0114,0.0074	0.68	−0.0028	−0.0132,0.0076	0.60	**−0.0596**	**−0.0935,−0.0256**	**0.001**
Life satisfaction	−0.0005	−0.0041,0.0032	0.79	−0.0008	−0.0052,0.0035	0.70	**−0.0138**	**−0.0268,−0.0009**	**0.036**
Self-realisation	**−0.0144**	**−0.0219,−0.0069**	**<0.001**	**−0.0146**	**−0.0231,−0.0060**	**0.001**	**−0.0691**	**−0.0950,−0.0432**	**<0.001**
Control-autonomy	**−0.0099**	**−0.0165,−0.0033**	**0.004**	**−0.0116**	**−0.0205,−0.0026**	**0.012**	**−0.0413**	**−0.0711,−0.0115**	**0.007**

*Additionally adjusted for mental ill-health*									
Positive affect	0.0007	−0.0089,0.0104	0.88	−0.0001	−0.0106,0.0103	0.98	**−0.0564**	**−0.0910,−0.0218**	**0.002**
Life satisfaction	0.0006	−0.0030,0.0043	0.73	0.0003	−0.0040,0.0047	0.89	−0.0118	−0.0251,0.0015	0.083
Self-realisation	**−0.0132**	**−0.0210,−0.0054**	**0.001**	**−0.0133**	**−0.0221,−0.0045**	**0.003**	**−0.0683**	**−0.0947,−0.0419**	**<0.001**
Control-autonomy	**−0.0083**	**−0.0150,−0.0016**	**0.016**	**−0.01**	**−0.0189,−0.0011**	**0.028**	**−0.0381**	**−0.0693,−0.0069**	**0.017**

*Additionally adjusted for covariates*									
Positive affect	0.0042	−0.0052,0.0136	0.38	0.0041	−0.0061,0.0143	0.43	**−0.0378**	**−0.0715,−0.0041**	**0.028**
Life satisfaction	0.0026	−0.0009,0.0061	0.14	0.0022	−0.0022,0.0065	0.32	−0.0043	−0.0176,0.0090	0.53
Self-realisation	**−0.0078**	**−0.0151,−0.0004**	**0.039**	−0.0077	−0.0167,0.0014	0.096	**−0.0476**	**−0.0735,−0.0218**	**<0.001**
Control-autonomy	−0.0039	−0.0107,0.0030	0.27	−0.0054	−0.0144,0.0037	0.24	−0.0214	−0.0520,0.0091	0.17

WOMEN (n = 4831)									
*Adjusted for time-constant factors*									
Positive affect	−0.0056	−0.0142,0.0029	. 19	−0.0086	−0.0175,0.0003	0.058	−0.0525	**−0.0824,−0.0226**	**0.001**
Life satisfaction	−0.0018	−0.0049,0.0013	0.24	−0.0022	−0.0054,0.0010	0.18	−0.014	**−0.0250,−0.0029**	**0.013**
Self-realisation	**−0.014**	**−0.0196,−0.0085**	**<0.001**	**−0.0149**	**−0.0213,−0.0085**	**<0.001**	**−0.0612**	**−0.0842,−0.0382**	**<0.001**
Control-autonomy	**−0.0095**	**−0.0156,−0.0035**	**0.002**	**−0.0126**	**−0.0194,−0.0057**	**<0.001**	**−0.0295**	**−0.0541,−0.0050**	**0.019**

*Additionally adjusted for mental ill-health*									
Positive affect	−0.0018	−0.0106,0.0071	0.69	−0.0046	−0.0135,0.0043	0.31	**−0.0463**	**−0.0775,−0.0152**	**0.004**
Life satisfaction	−0.0004	−0.0036,0.0029	0.82	−0.0006	−0.0040,0.0027	0.72	−0.0112	−0.0229,0.0004	0.059
Self-realisation	**−0.0121**	**−0.0179,−0.0063**	**<0.001**	**−0.0125**	**−0.0190,−0.0060**	**<0.001**	**−0.0583**	**−0.0838,−0.0329**	**<0.001**
Control-autonomy	**−0.0071**	**−0.0134,−0.0007**	**0.029**	**−0.0099**	**−0.0167,−0.0032**	**0.004**	−0.0234	−0.0495,0.0027	0.078

*Additionally adjusted for covariates*									
Positive affect	0.0005	−0.0081,0.0091	0.91	−0.0014	−0.0101,0.0073	0.75	**−0.0308**	**−0.0611,−0.0006**	**0.046**
Life satisfaction	0.0012	−0.0018,0.0043	0.43	0.0009	−0.0024,0.0042	0.58	−0.0053	−0.0164,0.0058	0.35
Self-realisation	**−0.0072**	**−0.0131,−0.0014**	**0.016**	**−0.0078**	**−0.0142,−0.0015**	**0.016**	**−0.0409**	**−0.0660,−0.0157**	**0.002**
Control-autonomy	−0.0027	−0.0091,0.0037	0.40	−0.0059	−0.0128,0.0010	0.093	−0.0083	−0.0335,0.0169	0.51

WBC: white blood cell count; Unadjusted models automatically account for all factors that are time-constant in older age, including age, sex, ethnicity, education, socio-economic status, genetics, past history of mental illness, and past history of wellbeing. Mental ill health includes depression and other psychiatric conditions. Additional covariates include chronic conditions, BMI and smoking. Boldface indicates p < .05

## 4 Discussion

This study found longitudinal associations between hedonic wellbeing and WBC, and between eudemonic wellbeing and WBC, CRP and fibrinogen. Increases in hedonic wellbeing were associated with decreases in WBC, while increases in eudemonic wellbeing were associated with decreases in WBC, CRP and fibrinogen. Notably, these associations were all independent of mental ill health, but partly explained by considering health-related factors such as BMI, smoking and sedentary behaviours. This relationship was present across both men and women, but was stronger in men. This is at odds with a previous cross-sectional study that found stronger associations between wellbeing and CRP and fibrinogen for women than men (Steptoe et al., 2012), but echoes previous research on depression and inflammation, which found stronger relationship between the two in men (Liukkonen et al., 2006, Penninx et al., 2003). It was also present across both those aged 50–65 and those aged over 65. This echoes a previous small-scale study suggesting the relationship between wellbeing and inflammation is stronger in those who are older (Ryff et al., 2004).

Our results build on previous cross-sectional research on wellbeing and inflammation by showing a longitudinal relationship between changes in wellbeing and changes in depression. These findings echo some previous cross-sectional findings showing associations between eudemonic wellbeing and CRP, fibrinogen, and WBC (Steptoe et al., 2012, Steptoe and Fancourt, 2019). However, it is notable that for hedonic wellbeing we only found associations with WBC. This goes against some previous work which showed cross-sectional associations between life satisfaction, CRP and fibrinogen, independent of depressive symptoms (Hamer and Chida, 2011), but is supported by a previous small study that found that eudemonic but not hedonic wellbeing was associated with inflammation (sIL-6r) (Ryff et al., 2004). In considering why we found associations between hedonic wellbeing and just WBC, it is noted that CRP and fibrinogen differ from WBC in being acute-phase hepatic proteins. Whilst inflammatory responses are bi-directionally interlinked, for example through WBC leading to the release of inflammatory interleukins, which can trigger hepatic release of CRP and fibrinogen (Leng et al., 2005, Wong et al., 2007), it is possible that, when analysing changes over long periods of time, there are more direct pathways between hedonic wellbeing and leukocytes than between hedonic wellbeing and acute-phase proteins. This finding also suggests that flourishing in life may have a broader association with inflammation than positive affect. But this remains to be explored further.

It is also of note that some of our results were explained by adjusting for health-related factors such as smoking and BMI. In literature on depression and inflammation, it has been proposed that depressive symptoms may lead to increased likelihood of smoking, with tobacco and nicotine in turn linked with a range of inflammatory markers (Shiels et al., 2014), so similar mechanisms could be at play for low wellbeing and smoking. Similarly, depressive symptoms have been found to promote weight accumulation (Miller et al., 2002), so it is possible that low wellbeing also promotes increases in weight. Weight in turn activates inflammatory responses through (i) increasing adipose tissue which can trigger an increase in IL-6 (Kern et al., 2001, Mohamed-Ali et al., 1997), and (ii) through increasing leptin release into circulation (Miller et al., 2003), which binds to receptors on WBC and upregulates IL-6. IL-6 can in turn stimulate hepatic release of CRP, thereby leading to wider inflammatory response (Gabay and Kushner, 1999, Yudkin et al., 2000). In our results, it is notable that although the relationship was present in those of a healthy BMI and those who were overweight obese, it was stronger in those who were overweight or obese, suggesting that biological pathways relating to weight could be key to the relationship shown.

This study has a number of strengths. First, we used data from a large nationally representative sample with multiple measurements over an 8-year period. Second, we moved beyond cross-sectional associations by showing associations between changes in wellbeing and inflammation. Our use of fixed effects modelling meant that all possible time-constant confounders were automatically accounted for, even if unobserved, reducing the risk of a spurious association. Additionally, we explored whether associations between wellbeing and inflammation were explained by depression. Further, we used two different measures of both hedonic and eudemonic wellbeing. The consistency of the results between each pair of measures suggests that our findings are not merely an artefact of our specific measurement. Finally, our use of three different measures of inflammation helps to avoid the possibility of false negative findings, which has previously been discussed as a challenge in studies of inflammation (Pariante, 2019).

However, this study also has several limitations. First, we only had access to longitudinal data on three inflammatory biomarkers within ELSA, and so although our findings have shown relative consistency, indicating perhaps a general inflammatory response especially in relation to eudemonic wellbeing, whether wellbeing is independently associated with other markers of inflammation such as cytokine response remains unknown. Second, we only had two measures of hedonic wellbeing and two measures of eudemonic wellbeing. In particular for eudemonic wellbeing, theoretical literature suggests there are a wide range of different components that may have different associations with health (Kashdan et al., 2008). Therefore, future studies could extend the findings here by focusing on other related positive psychological measures such as vitality, self-esteem and personal growth. Third, our analyses explored associations between changes in wellbeing and biological markers, but this study did not attempt to identify the direction of causality. Future research is needed to elucidate whether changes in wellbeing drive changes in inflammation or vice versa, or whether, as for depression, this relationship is bi-directional (Amodeo et al., 2017). Fourth, we did not have data on whether participants were on anti-inflammatory or psychotropic medications. If an individual was on these consistently across the three waves, then the effects would have automatically been accounted for under the fixed effects model. However, if an individual started or stopped a new course of medication during the 8-year period, then the effects of this on the findings remain unknown. Fifth, we used multiple imputation to account for missing data, in particular to impute missing WBC data in wave 2. Notably, our un-imputed results showed comparable findings. Nevertheless, the correct treatment of missing data in biological studies remains a challenge. Finally, this study focused exclusively on adults aged 50+. However, our results suggested that there was moderation by age. Although the differences in results between those aged 50–65 and over 65 were very small, future studies could explore whether there are more marked differences when comparing with younger cohorts.

In conclusion, this study builds on the strong literature showing a relationship between mental ill health and inflammation by showing that there is also an apparently independent relationship between mental wellbeing and inflammation that is unexplained by socio-economic factors or other time-constant factors. Although this relationship is partly explained by health-related factors such as BMI and chronic conditions, for WBC in particular, the relationship appears to persist independent of this, and is particularly strong for eudemonic aspects of wellbeing. This study did not attempt to disentangle the direction of effect, but if the relationship between inflammation and wellbeing resembles that of inflammation and depression, we might hypothesise that this is bidirectional. Therefore, future studies could explore whether interventions to increase wellbeing can help causally to reduce levels of inflammation, and thereby reduce the risk of developing inflammatory conditions.

## Declaration of Competing Interest

The authors declare that they have no known competing financial interests or personal relationships that could have appeared to influence the work reported in this paper.

## References

[b0005] Allison P. (2009). Fixed Effects Regression Models.

[b0010] Amodeo G., Trusso M.A., Fagiolini A. (2017). Depression and inflammation: disentangling a clear yet complex and multifaceted link. Neuropsychiatry.

[b0015] Aschbacher K., Epel E., Wolkowitz O.M., Prather A.A., Puterman E., Dhabhar F.S. (2012). Maintenance of a positive outlook during acute stress protects against pro-inflammatory reactivity and future depressive symptoms. Brain. Behav. Immun..

[b0020] Bartels M., Saviouk V., De Moor M.H., Willemsen G., van Beijsterveldt T.C., Hottenga J.-J., De Geus E.J., Boomsma D.I. (2010). Heritability and genome-wide linkage scan of subjective happiness. Twin Res. Hum. Genet..

[b0025] Blevins C.L., Sagui S.J., Bennett J.M. (2017). Inflammation and positive affect: examining the stress-buffering hypothesis with data from the National Longitudinal Study of Adolescent to Adult Health. Brain. Behav. Immun..

[b0030] Brouwers C., Mommersteeg P.M.C., Nyklíček I., Pelle A.J., Westerhuis B.L., Szabó B.M., Denollet J. (2013). Positive affect dimensions and their association with inflammatory biomarkers in patients with chronic heart failure. Biol. Psychol..

[b0035] Dahl J., Ormstad H., Aass H.C.D., Malt U.F., Bendz L.T., Sandvik L., Brundin L., Andreassen O.A. (2014). The plasma levels of various cytokines are increased during ongoing depression and are reduced to normal levels after recovery. Psychoneuroendocrinology.

[b0040] Diener E., Emmons R.A., Larsen R.J., Griffin S. (1985). The satisfaction with life scale. J. Pers. Assess..

[b0045] Dowlati Y., Herrmann N., Swardfager W., Liu H., Sham L., Reim E.K., Lanctôt K.L. (2010). A meta-analysis of cytokines in major depression. Biol. Psychiatry.

[b0050] Franz C.E., Panizzon M.S., Eaves L.J., Thompson W., Lyons M.J., Jacobson K.C., Tsuang M., Glatt S.J., Kremen W.S. (2012). Genetic and environmental multidimensionality of well-and ill-being in middle aged twin men. Behav. Genet..

[b0055] Gabay C., Kushner I. (1999). Acute-phase proteins and other systemic responses to inflammation. N. Engl. J. Med..

[b0060] Graig R., Deverill C., Pickering K. (2004). Quality control of blood, saliva and urine analytes. Health Surv. Engl..

[b0065] Hamer M., Chida Y. (2011). Life satisfaction and inflammatory biomarkers: the 2008 Scottish Health Survey1. Jpn. Psychol. Res..

[b0070] Ironson G., Banerjee N., Fitch C., Krause N. (2018). Positive emotional well-being, health behaviors, and inflammation measured by C-Reactive protein. Soc. Sci. Med..

[b0075] Kashdan T.B., Biswas-Diener R., King L.A. (2008). Reconsidering happiness: the costs of distinguishing between hedonics and eudaimonia. J. Posit. Psychol..

[b0080] Kendler K.S., Myers J.M., Maes H.H., Keyes C.L. (2011). The relationship between the genetic and environmental influences on common internalizing psychiatric disorders and mental well-being. Behav. Genet..

[b0085] Kern P.A., Ranganathan S., Li C., Wood L., Ranganathan G. (2001). Adipose tissue tumor necrosis factor and interleukin-6 expression in human obesity and insulin resistance. Am. J. Physiol.-Endocrinol. Metab..

[b0090] Keyes C.L. (2005). Mental illness and/or mental health? Investigating axioms of the complete state model of health. J. Consult. Clin. Psychol..

[b0095] Kim D., Kubzansky L.D., Baccarelli A., Sparrow D., Spiro A., Tarantini L., Cantone L., Vokonas P., Schwartz J. (2016). Psychological factors and DNA methylation of genes related to immune/inflammatory system markers: the VA Normative Aging Study. BMJ Open.

[b0100] Leng S., Xue Q.-L., Huang Y., Semba R., Chaves P., Bandeen-Roche K., Fried L., Walston J. (2005). Total and Differential white blood cell counts and their associations with circulating interleukin-6 levels in community-dwelling older women. J. Gerontol. Ser. A.

[b0105] Liukkonen T., Silvennoinen-Kassinen S., Jokelainen J., Räsänen P., Leinonen M., Meyer-Rochow V.B., Timonen M. (2006). The association between C-reactive protein levels and depression: results from the northern Finland 1966 birth cohort study. Biol. Psychiatry.

[b0110] Miller G.E., Freedland K.E., Carney R.M., Stetler C.A., Banks W.A. (2003). Pathways linking depression, adiposity, and inflammatory markers in healthy young adults. Brain. Behav. Immun..

[b0115] Miller G.E., Stetler C.A., Carney R.M., Freedland K.E., Banks W.A. (2002). Clinical depression and inflammatory risk markers for coronary heart disease. Am. J. Cardiol..

[b0120] Mohamed-Ali V., Goodrick S., Rawesh A., Katz D.R., Miles J.M., Yudkin J.S., Klein S., Coppack S.W. (1997). Subcutaneous adipose tissue releases interleukin-6, but not tumor necrosis factor-α, in vivo. J. Clin. Endocrinol. Metab..

[b0125] Moons K.G.M., Donders R.A.R.T., Stijnen T., Harrell F.E. (2006). Using the outcome for imputation of missing predictor values was preferred. J. Clin. Epidemiol..

[b0130] Moreno P.I., Moskowitz A.L., Ganz P.A., Bower J.E. (2016). Positive affect and inflammatory activity in breast cancer survivors: examining the role of affective arousal. Psychosom. Med..

[b0135] Okbay A., Baselmans B.M.L., De Neve J.-E., Turley P., Nivard M.G., Fontana M.A., Meddens S.F.W., Linnér R.K., Rietveld C.A., Derringer J., Gratten J., Lee J.J., Liu J.Z., de Vlaming R., Ahluwalia T.S., Buchwald J., Cavadino A., Frazier-Wood A.C., Furlotte N.A., Garfield V., Geisel M.H., Gonzalez J.R., Haitjema S., Karlsson R., van der Laan S.W., Ladwig K.-H., Lahti J., van der Lee S.J., Lind P.A., Liu T., Matteson L., Mihailov E., Miller M.B., Minica C.C., Nolte I.M., Mook-Kanamori D., van der Most P.J., Oldmeadow C., Qian Y., Raitakari O., Rawal R., Realo A., Rueedi R., Schmidt B., Smith A.V., Stergiakouli E., Tanaka T., Taylor K., Thorleifsson G., Wedenoja J., Wellmann J., Westra H.-J., Willems S.M., Zhao W., LifeLines Cohort Study, Amin N., Bakshi A., Bergmann S., Bjornsdottir G., Boyle P.A., Cherney S., Cox S.R., Davies G., Davis O.S.P., Ding J., Direk N., Eibich P., Emeny R.T., Fatemifar G., Faul J.D., Ferrucci L., Forstner A.J., Gieger C., Gupta R., Harris T.B., Harris J.M., Holliday E.G., Hottenga J.-J., De Jager P.L., Kaakinen M.A., Kajantie E., Karhunen V., Kolcic I., Kumari M., Launer L.J., Franke L., Li-Gao R., Liewald D.C., Koini M., Loukola A., Marques-Vidal P., Montgomery G.W., Mosing M.A., Paternoster L., Pattie A., Petrovic K.E., Pulkki-Råback L., Quaye L., Räikkönen K., Rudan I., Scott R.J., Smith J.A., Sutin A.R., Trzaskowski M., Vinkhuyzen A.E., Yu L., Zabaneh D., Attia J.R., Bennett D.A., Berger K., Bertram L., Boomsma D.I., Snieder H., Chang S.-C., Cucca F., Deary I.J., van Duijn C.M., Eriksson J.G., Bültmann U., de Geus E.J.C., Groenen P.J.F., Gudnason V., Hansen T., Hartman C.A., Haworth C.M.A., Hayward C., Heath A.C., Hinds D.A., Hyppönen E., Iacono W.G., Järvelin M.-R., Jöckel K.-H., Kaprio J., Kardia S.L.R., Keltikangas-Järvinen L., Kraft P., Kubzansky L.D., Lehtimäki T., Magnusson P.K.E., Martin N.G., McGue M., Metspalu A., Mills M., de Mutsert R., Oldehinkel A.J., Pasterkamp G., Pedersen N.L., Plomin R., Polasek O., Power C., Rich S.S., Rosendaal F.R., den Ruijter H.M., Schlessinger D., Schmidt H., Svento R., Schmidt R., Alizadeh B.Z., Sørensen T.I.A., Spector T.D., Starr J.M., Stefansson K., Steptoe A., Terracciano A., Thorsteinsdottir U., Thurik A.R., Timpson N.J., Tiemeier H., Uitterlinden A.G., Vollenweider P., Wagner G.G., Weir D.R., Yang J., Conley D.C., Smith G.D., Hofman A., Johannesson M., Laibson D.I., Medland S.E., Meyer M.N., Pickrell J.K., Esko T., Krueger R.F., Beauchamp J.P., Koellinger P.D., Benjamin D.J., Bartels M., Cesarini D. (2016). Genetic variants associated with subjective well-being, depressive symptoms, and neuroticism identified through genome-wide analyses. Nat. Genet..

[b0140] Okely J.A., Weiss A., Gale C.R. (2017). Well-being and arthritis incidence: the role of inflammatory mechanisms. findings from the english longitudinal study of ageing. Psychosom. Med..

[b0145] Ong A.D., Benson L., Zautra A.J., Ram N. (2018). Emodiversity and biomarkers of inflammation. Emotion.

[b0150] Pariante C.M. (2019). Did spider-man work in the NESDA cohort? In immunopsychiatry, with great power comes great responsibility. Biol. Psychiatry.

[b0155] Penninx B.W.J.H., Kritchevsky S.B., Yaffe K., Newman A.B., Simonsick E.M., Rubin S., Ferrucci L., Harris T., Pahor M. (2003). Inflammatory markers and depressed mood in older persons: results from the Health, Aging and Body Composition study. Biol. Psychiatry.

[b0160] Plomin R., Scheier M.F., Bergeman C.S., Pedersen N.L., Nesselroade J.R., McClearn G.E. (1992). Optimism, pessimism and mental health: a twin/adoption analysis. Personal. Individ. Differ..

[b0165] Radloff L.S. (1977). The CES-D Scale: a self-report depression scale for research in the general population. Appl. Psychol. Meas..

[b0170] Raison C.L., Miller A.H. (2013). Malaise, melancholia and madness: the evolutionary legacy of an inflammatory bias. Brain. Behav. Immun..

[b0175] Rosenkranz M.A., Jackson D.C., Dalton K.M., Dolski I., Ryff C.D., Singer B.H., Muller D., Kalin N.H., Davidson R.J. (2003). Affective style and in vivo immune response: neurobehavioral mechanisms. Proc. Natl. Acad. Sci..

[b0180] Ryff C.D., Love G.D., Urry H.L., Muller D., Rosenkranz M.A., Friedman E.M., Davidson R.J., Singer B. (2006). Psychological well-being and ill-being: do they have distinct or mirrored biological correlates?. Psychother. Psychosom..

[b0185] Ryff C.D., Singer B. (1998). The contours of positive human health. Psychol. Inq..

[b0190] Ryff C.D., Singer B.H., Dienberg Love G. (2004). Positive health: connecting well–being with biology. Philos. Trans. R. Soc. Lond. B. Biol. Sci..

[b0195] Shiels M.S., Katki H.A., Freedman N.D., Purdue M.P., Wentzensen N., Trabert B., Kitahara C.M., Furr M., Li Y., Kemp T.J., Goedert J.J., Chang C.M., Engels E.A., Caporaso N.E., Pinto L.A., Hildesheim A., Chaturvedi A.K. (2014). Cigarette smoking and variations in systemic immune and inflammation markers. JNCI J. Natl. Cancer Inst..

[b0200] Stellar J.E., John-Henderson N., Anderson C.L., Gordon A.M., McNeil G.D., Keltner D. (2015). Positive affect and markers of inflammation: discrete positive emotions predict lower levels of inflammatory cytokines. Emotion.

[b0205] Steptoe A., Breeze E., Banks J., Nazroo J. (2013). Cohort profile: the English longitudinal study of ageing. Int. J. Epidemiol..

[b0210] Steptoe A., Demakakos P., de Oliveira C., Wardle J. (2012). Distinctive biological correlates of positive psychological well-being in older men and women. Psychosom. Med..

[b0215] Steptoe A., Fancourt D. (2019). Leading a meaningful life at older ages and its relationship with social engagement, prosperity, health, biology, and time use. Proc. Natl. Acad. Sci..

[b0220] Steptoe A., O’Donnell K., Badrick E., Kumari M., Marmot M. (2008). Neuroendocrine and inflammatory factors associated with positive affect in healthy men and women: the Whitehall II study. Am. J. Epidemiol..

[b0225] Sterne J.A.C., White I.R., Carlin J.B., Spratt M., Royston P., Kenward M.G., Wood A.M., Carpenter J.R. (2009). Multiple imputation for missing data in epidemiological and clinical research: potential and pitfalls. BMJ.

[b0230] Uchino B.N., de Grey R.G.K., Cronan S., Smith T.W., Diener E., Joel S., Bosch J. (2018). Life satisfaction and inflammation in couples: an actor–partner analysis. J. Behav. Med..

[b0235] Urry H.L., Nitschke J.B., Dolski I., Jackson D.C., Dalton K.M., Mueller C.J., Rosenkranz M.A., Ryff C.D., Singer B.H., Davidson R.J. (2004). Making a life worth living: neural correlates of well-being. Psychol. Sci..

[b0240] Vanhoutte B. (2014). The multidimensional structure of subjective well-being in later life. J. Popul. Ageing.

[b0245] Vanhoutte, B., 2012. Measuring subjective well-being: a review [WWW Document]. CCSR Woking Pap. Manch. CCSR Univ. Manch. 2012 Work. Pap. No 2012-06. URL https://www.escholar.manchester.ac.uk/uk-ac-man-scw:195190 (accessed 10.17.17).

[b0250] Wong L.Y.F., Leung R.Y.H., Ong K.L., Cheung B.M.Y. (2007). Plasma levels of fibrinogen and C-reactive protein are related to interleukin-6 gene -572C>G polymorphism in subjects with and without hypertension. J. Hum. Hypertens..

[b0255] Yudkin J.S., Kumari M., Humphries S.E., Mohamed-Ali V. (2000). Inflammation, obesity, stress and coronary heart disease: is interleukin-6 the link?. Atherosclerosis.

