# Supplementary material for: The longitudinal relationship between changes in wellbeing and inflammatory markers: Are associations independent of depression?
Source: Brain Behav Immun. 2020 Jan;83:146–52. doi: 10.1016/j.bbi.2019.10.004 (PMC6928572; doi:10.1016/j.bbi.2019.10.004)
Supplement: Supplementary Data 1 [file mmc1.docx]

**Supplementary Table 1: Fixed effects models showing associations between changes in wellbeing and changes in inflammation: using depression as a continuous rather than binary variable**

|  | CRP | |  | Fibrinogen | |  | WBC | |  |
| --- | --- | --- | --- | --- | --- | --- | --- | --- | --- |
|  | Coef | 95% CI | p | Coef | 95% CI | p | Coef | 95% CI | p |
| Adjusted for time-constant factors | | | | | |  |  |  |  |
| Positive affect | -0.004 | -0.0108,0.0029 | .25 | -0.0059 | -0.0127,0.0010 | .094 | **-0.0558** | **-0.0799,-0.0317** | **<.001** |
| Life satisfaction | -0.0012 | -0.0037,0.0012 | .32 | -0.0016 | -0.0044,0.0012 | .26 | **-0.0139** | **-0.0233,-0.0046** | **.004** |
| Self-realisation | **-0.0142** | **-0.0190,-0.0094** | **<.001** | **-0.0147** | **-0.0200,-0.0095** | **<.001** | **-0.0648** | **-0.0835,-0.0461** | **<.001** |
| Control-autonomy | **-0.0097** | **-0.0145,-0.0049** | **<.001** | **-0.0121** | **-0.0179,-0.0063** | **<.001** | **-0.0348** | **-0.0561,-0.0135** | **.002** |
| Additionally adjusted for mental ill-health | | | | | |  |  |  |  |
| Positive affect | 0.0025 | -0.0048,0.0097 | .50 | 0.001 | -0.0060,0.0079 | **.78** | **-0.0443** | **-0.0711,-0.0174** | **.001** |
| Life satisfaction | 0.0013 | -0.0013,0.0040 | .32 | 0.0012 | -0.0017,0.0042 | .41 | -0.0083 | -0.0184,0.0018 | .11 |
| Self-realisation | **-0.0109** | **-0.0161,-0.0056** | **<.001** | **-0.0107** | **-0.0163,-0.0051** | **<.001** | **-0.0595** | **-0.0811,-0.0379** | **<.001** |
| Control-autonomy | **-0.0056** | **-0.0109,-0.0002** | **.043** | **-0.0077** | **-0.0138,-0.0016** | **.014** | -0.0239 | -0.0484,0.0005 | .055 |
| Additionally adjusted for covariates | | | | | |  |  |  |  |
| Positive affect | 0.0023 | -0.0048,0.0093 | .53 | 0.0013 | -0.0054,0.0080 | .70 | **-0.0342** | **-0.0590,-0.0093** | **.008** |
| Life satisfaction | 0.0019 | -0.0006,0.0043 | .14 | 0.0015 | -0.0014,0.0044 | .30 | -0.0048 | -0.0146,0.0049 | .33 |
| Self-realisation | **-0.0074** | **-0.0125,-0.0024** | **.004** | **-0.0076** | **-0.0130,-0.0022** | **.006** | **-0.0439** | **-0.0640,-0.0238** | **<.001** |
| Control-autonomy | -0.0032 | -0.0085,0.0020 | .23 | -0.0056 | -0.0115,0.0002 | .060 | -0.0142 | -0.0369,0.0084 | .22 |

*WBC: white blood cell count; Unadjusted models automatically account for all factors that are time-constant in older age, including age, sex, ethnicity, education, socio-economic status, genetics, past history of mental illness, and past history of wellbeing. Mental ill health includes depression and other psychiatric conditions. Additional covariates include chronic conditions, BMI and smoking. Boldface indicates p<.05*

**Supplementary Table 2: Fixed effects models showing associations between changes in wellbeing and changes in inflammation: excluding individuals who had an infection in the 3 weeks prior to blood sampling**

| N=22,032 | CRP | |  | Fibrinogen | | |  | WBC | |  |
| --- | --- | --- | --- | --- | --- | --- | --- | --- | --- | --- |
|  | Coef | 95% CI | p | Coef | 95% CI | p | | Coef | 95% CI | p |
| Adjusted for time-constant factors | | | | | |  | |  |  |  |
| Positive affect | -0.0047 | -0.0119,0.0024 | .19 | **-0.008** | **-0.0153,-0.0006** | **.034** | | **-0.0569** | **-0.0826,-0.0311** | **<.001** |
| Life satisfaction | -0.0018 | -0.0045,0.0009 | .18 | -0.002 | -0.0051,0.0010 | .19 | | **-0.0133** | **-0.0230,-0.0035** | **.008** |
| Self-realisation | **-0.015** | **-0.0203,-0.0096** | **<.001** | **-0.0162** | **-0.0217,-0.0108** | **<.001** | | **-0.0646** | **-0.0832,-0.0460** | **<.001** |
| Control-autonomy | **-0.0104** | **-0.0157,-0.0051** | **<.001** | **-0.0129** | **-0.0192,-0.0067** | **<.001** | | **-0.0357** | **-0.0588,-0.0126** | **.003** |
| Additionally adjusted for mental ill-health | | | | | |  | |  |  |  |
| Positive affect | -0.0013 | -0.0088,0.0061 | .72 | -0.0046 | -0.0118,0.0027 | .22 | | **-0.0518** | **-0.0788,-0.0249** | **<.001** |
| Life satisfaction | -0.0005 | -0.0033,0.0023 | .71 | -0.0006 | -0.0038,0.0025 | .69 | | **-0.0107** | **-0.0211,-0.0003** | **.045** |
| Self-realisation | **-0.0134** | **-0.0189,-0.0078** | **<.001** | **-0.0144** | **-0.0199,-0.0088** | **<.001** | | **-0.0624** | **-0.0827,-0.0421** | **<.001** |
| Control-autonomy | **-0.0083** | **-0.0138,-0.0028** | **.003** | **-0.0107** | **-0.0170,-0.0045** | **.001** | | **-0.0307** | **-0.0562,-0.0051** | **.019** |
| Additionally adjusted for covariates | | | | | |  | |  |  |  |
| Positive affect | 0.0036 | -0.0035,0.0107 | .32 | 0.0013 | -0.0058,0.0085 | .72 | | **-0.0306** | **-0.0568,-0.0044** | **.023** |
| Life satisfaction | 0.002 | -0.0006,0.0047 | .13 | 0.0018 | -0.0013,0.0049 | .25 | | -0.0029 | -0.0131,0.0073 | .57 |
| Self-realisation | -0.0038 | -0.0093,0.0017 | .17 | -0.0034 | -0.0092,0.0024 | .24 | | **-0.0333** | **-0.0533,-0.0133** | **.001** |
| Control-autonomy | -0.0006 | -0.0062,0.0050 | .83 | -0.0026 | -0.0090,0.0039 | .43 | | **-0.0077** | **-0.0329,0.0175** | .55 |

*WBC: white blood cell count; Unadjusted models automatically account for all factors that are time-constant in older age, including age, sex, ethnicity, education, socio-economic status, genetics, past history of mental illness, and past history of wellbeing. Mental ill health includes depression and other psychiatric conditions. Additional covariates include chronic conditions, BMI and smoking. Boldface indicates p<.05.*

**Supplementary Table 3: Fixed effects models showing associations between changes in wellbeing and changes in WBC, excluding wave 2 where WBC count was not measured and therefore imputed**

|  | WBC | | |
| --- | --- | --- | --- |
|  | Coef | 95% CI | p |
| Adjusted for time-constant factors | | | |
| Positive affect | **-0.0485** | **-0.0807,-0.0162** | **.003** |
| Life satisfaction | -0.0111 | -0.0227,0.0004 | .059 |
| Self-realisation | **-0.0612** | **-0.0848,-0.0376** | **<.001** |
| Control-autonomy | **-0.0352** | **-0.0621,-0.0083** | **.011** |
| Additionally adjusted for mental ill-health | | | |
| Positive affect | **-0.0447** | **-0.0768,-0.0126** | **.007** |
| Life satisfaction | -0.0092 | -0.0211,0.0028 | .13 |
| Self-realisation | **-0.06** | **-0.0841,-0.0360** | **<.001** |
| Control-autonomy | **-0.0314** | **-0.0596,-0.0033** | **.029** |
| Additionally adjusted for covariates | | | |
| Positive affect | -0.0243 | -0.0553,0.0067 | .12 |
| Life satisfaction | -0.0026 | -0.0141,0.0089 | .66 |
| Self-realisation | **-0.0312** | **-0.0552,-0.0072** | **.011** |
| Control-autonomy | -0.0098 | -0.0363,0.0166 | .46 |

*WBC: white blood cell count; Unadjusted models automatically account for all factors that are time-constant in older age, including age, sex, ethnicity, education, socio-economic status, genetics, past history of mental illness, and past history of wellbeing. Mental ill health includes depression and other psychiatric conditions. Additional covariates include chronic conditions, BMI and smoking. Boldface indicates p<.05.*

**Supplementary Table 4: Fixed effects models showing associations between changes in wellbeing and changes in inflammation: split by age**

|  | CRP | | |  | | Fibrinogen | |  | | WBC | | |  |
| --- | --- | --- | --- | --- | --- | --- | --- | --- | --- | --- | --- | --- | --- |
|  | Coef | 95% CI | p | | Coef | | 95% CI | p | | Coef | | 95% CI | p |
| UNDER 65 (N=4,341) | | | | | | | |  |  | |  | |  |
| Adjusted for time-constant factors | | | | | | | |  |  | |  | |  |
| Positive affect | -0.0043 | -0.0130,0.0045 | .34 | | -0.0052 | | -0.0149,0.0045 | **.29** | | **-0.0515** | | **-0.0862,-0.0168** | **.004** |
| Life satisfaction | -0.0014 | -0.0047,0.0020 | .42 | | -0.0013 | | -0.0050,0.0024 | **.49** | | -0.0119 | | -0.0240,0.0002 | **.055** |
| Self-realisation | **-0.0133** | **-0.0199,-0.0068** | **<.001** | | **-0.0117** | | **-0.0189,-0.0045** | **.002** | | **-0.0552** | | **-0.0803,-0.0302** | **<.001** |
| Control-autonomy | **-0.0085** | **-0.0156,-0.0015** | **.018** | | **-0.0093** | | **-0.0169,-0.0016** | **.018** | | -0.0223 | | -0.0505,0.0058 | .12 |
| Additionally adjusted for mental ill-health | | | | | | | |  |  | |  | |  |
| Positive affect | -0.0008 | -0.0099,0.0084 | .87 | | -0.002 | | -0.0118,0.0078 | **.69** | | **-0.0491** | | **-0.0856,-0.0126** | **.009** |
| Life satisfaction | -0.0001 | -0.0036,0.0035 | .96 | | 0.00 | | -0.0039,0.0039 | .99 | | -0.0103 | | -0.0233,0.0026 | .12 |
| Self-realisation | **-0.0116** | **-0.0184,-0.0047** | **.001** | | **-0.0098** | | **-0.0171,-0.0025** | **.009** | | **-0.0544** | | **-0.0813,-0.0276** | **<.001** |
| Control-autonomy | -0.0065 | -0.0137,0.0007 | .077 | | -0.0073 | | -0.0149,0.0003 | .059 | | -0.019 | | -0.0488,0.0108 | .21 |
| Additionally adjusted for covariates | | | | | | | |  |  | |  | |  |
| Positive affect | 0.0022 | -0.0069,0.0113 | .63 | | 0.0016 | | -0.0082,0.0113 | .75 | | -0.032 | | -0.0677,0.0037 | **.**078 |
| Life satisfaction | 0.0015 | -0.0019,0.0048 | .38 | | 0.0013 | | -0.0025,0.0052 | .50 | | -0.0047 | | -0.0175,0.0081 | .47 |
| Self-realisation | -0.0064 | -0.0130,0.0001 | .055 | | -0.0053 | | -0.0128,0.0021 | .16 | | **-0.0367** | | **-0.0629,-0.0104** | **.006** |
| Control-autonomy | -0.0022 | -0.0094,0.0049 | .54 | | -0.0038 | | -0.0115,0.0040 | .34 | | -0.0046 | | -0.0331,0.0240 | .75 |
| 65 AND ABOVE (N=4,439) | | | | | | | |  |  | |  | |  |
| Adjusted for time-constant factors | | | | | | | |  |  | |  | |  |
| Positive affect | -0.0038 | -0.0127,0.0051 | .40 | | -0.0066 | | -0.0161,0.0030 | **.18** | | **-0.0593** | | **-0.0897,-0.0290** | **<.001** |
| Life satisfaction | -0.0013 | -0.0044,0.0019 | .43 | | -0.0021 | | -0.0058,0.0016 | **.26** | | **-0.0158** | | **-0.0282,-0.0035** | **.012** |
| Self-realisation | **-0.0147** | **-0.0208,-0.0086** | **<.001** | | **-0.0165** | | **-0.0232,-0.0098** | **<.001** | | **-0.0712** | | **-0.0932,-0.0492** | **<.001** |
| Control-autonomy | **-0.0107** | **-0.0167,-0.0047** | **.001** | | **-0.0145** | | **-0.0220,-0.0070** | **<.001** | | **-0.0443** | | **-0.0686,-0.0200** | **<.001** |
| Additionally adjusted for mental ill-health | | | | | | | |  |  | |  | |  |
| Positive affect | -0.0006 | -0.0098,0.0086 | .90 | | -0.0029 | | -0.0125,0.0066 | **.54** | | **-0.0528** | | **-0.0837,-0.0218** | **.001** |
| Life satisfaction | 0.0001 | -0.0032,0.0033 | .97 | | -0.0006 | | -0.0043,0.0031 | .74 | | **-0.0127** | | **-0.0253,-0.0001** | **.048** |
| Self-realisation | **-0.0132** | **-0.0195,-0.0068** | **<.001** | | **-0.0146** | | **-0.0215,-0.0076** | **<.001** | | **-0.0684** | | **-0.0916,-0.0451** | **<.001** |
| Control-autonomy | **-0.0086** | **-0.0149,-0.0023** | **.008** | | **-0.0122** | | **-0.0197,-0.0046** | **.002** | | **-0.0384** | | **-0.0649,-0.0120** | **.005** |
| Additionally adjusted for covariates | | | | | | | |  |  | |  | |  |
| Positive affect | 0.0022 | -0.0066,0.0110 | .63 | | 0.0008 | | -0.0082,0.0099 | .85 | | **-0.036** | | **-0.0661,-0.0058** | **.02** |
| Life satisfaction | 0.002 | -0.0011,0.0051 | .20 | | 0.0013 | | -0.0023,0.0049 | .48 | | -0.0053 | | -0.0177,0.0070 | .40 |
| Self-realisation | **-0.008** | **-0.0145,-0.0015** | **.016** | | **-0.009** | | **-0.0159,-0.0021** | **.011** | | **-0.0483** | | **-0.0719,-0.0247** | **<.001** |
| Control-autonomy | -0.0041 | -0.0108,0.0025 | .22 | | -0.0075 | | -0.0152,0.0003 | .059 | | -0.0216 | | -0.0486,0.0054 | .12 |

*WBC: white blood cell count; Unadjusted models automatically account for all factors that are time-constant in older age, including age, sex, ethnicity, education, socio-economic status, genetics, past history of mental illness, and past history of wellbeing. Mental ill health includes depression and other psychiatric conditions. Additional covariates include chronic conditions, BMI and smoking. Boldface indicates p<.05*

**Supplementary Table 5: Fixed effects models showing associations between changes in wellbeing and changes in inflammation: split by BMI**

|  | CRP | | |  | | Fibrinogen | |  | WBC | | | |  |
| --- | --- | --- | --- | --- | --- | --- | --- | --- | --- | --- | --- | --- | --- |
|  | Coef | 95% CI | p | | Coef | | 95% CI | p | | Coef | | 95% CI | p |
| BMI <25 (2,397) | | | | | | | |  |  | |  | |  |
| Adjusted for time-constant factors | | | | | | | |  |  | |  | |  |
| Positive affect | -0.0033 | -0.0145,0.0079 | .56 | | -0.005 | | -0.0184,0.0084 | .46 | | **-0.0627** | | **-0.1066,-0.0187** | **.006** |
| Life satisfaction | -0.001 | -0.0052,0.0031 | .62 | | -0.0018 | | -0.0065,0.0029 | .46 | | -0.014 | | -0.0313,0.0033 | .11 |
| Self-realisation | **-0.0125** | **-0.0200,-0.0050** | **.001** | | **-0.0136** | | **-0.0233,-0.0039** | **.006** | | **-0.0613** | | **-0.0935,-0.0290** | **<.001** |
| Control-autonomy | **-0.0085** | **-0.0168,-0.0003** | **.043** | | **-0.0138** | | **-0.0237,-0.0040** | **.006** | | -0.0356 | | -0.0718,0.0006 | .054 |
| Additionally adjusted for mental ill-health | | | | | | | |  |  | |  | |  |
| Positive affect | 0.0002 | -0.0116,0.0121 | .97 | | -0.0004 | | -0.0141,0.0134 | .96 | | **-0.0575** | | **-0.1009,-0.0140** | **.01** |
| Life satisfaction | 0.0003 | -0.0040,0.0047 | .89 | | 0.00 | | -0.0048,0.0048 | .99 | | -0.0114 | | -0.0290,0.0063 | .21 |
| Self-realisation | **-0.0107** | **-0.0188,-0.0027** | **.009** | | **-0.0109** | | **-0.0212,-0.0006** | **.039** | | **-0.0585** | | **-0.0916,-0.0254** | **.001** |
| Control-autonomy | -0.0064 | -0.0151,0.0023 | .15 | | -0.0111 | | **-0.0212,-0.0010** | **.032** | | -0.0305 | | -0.0674,0.0064 | .10 |
| Additionally adjusted for covariates | | | | | | | |  |  | |  | |  |
| Positive affect | 0.0027 | -0.0090,0.0144 | .65 | | 0.0035 | | -0.0103,0.0172 | .62 | | -0.04 | | -0.0819,0.0018 | **.**061 |
| Life satisfaction | 0.0016 | -0.0028,0.0059 | .48 | | 0.0017 | | -0.0032,0.0066 | .49 | | -0.0041 | | -0.0214,0.0131 | .64 |
| Self-realisation | -0.0071 | -0.0153,0.0011 | .088 | | -0.0058 | | -0.0166,0.0049 | .29 | | -**0.0402** | | **-0.0735,-0.0070** | **.018** |
| Control-autonomy | -0.0032 | -0.0119,0.0055 | .47 | | -0.0069 | | -0.0174,0.0035 | .19 | | -0.0152 | | -0.0514,0.0210 | .41 |
| BMI 25+ (N=6,361) | | | | | | | |  |  | |  | |  |
| Adjusted for time-constant factors | | | | | | | |  |  | |  | |  |
| Positive affect | -0.0042 | -0.0119,0.0035 | .28 | | -0.0062 | | -0.0150,0.0027 | .17 | | **-0.0532** | | **-0.0796,-0.0268** | **<.001** |
| Life satisfaction | -0.0013 | -0.0040,0.0013 | .32 | | -0.0016 | | -0.0048,0.0017 | .34 | | **-0.0138** | | **-0.0238,-0.0039** | **.007** |
| Self-realisation | **-0.015** | **-0.0206,-0.0094** | **<.001** | | **-0.0152** | | **-0.0213,-0.0091** | **<.001** | | **-0.0662** | | **-0.0865,-0.0459** | **<.001** |
| Control-autonomy | **-0.0102** | **-0.0156,-0.0047** | **<.001** | | **-0.0115** | | **-0.0183,-0.0047** | **.001** | | **-0.0343** | | **-0.0583,-0.0103** | **.006** |
| Additionally adjusted for mental ill-health | | | | | | | |  |  | |  | |  |
| Positive affect | -0.0009 | -0.0089,0.0071 | .83 | | -0.0031 | | -0.0120,0.0058 | **.**49 | | **-0.0488** | | **-0.0775,-0.0202** | **.001** |
| Life satisfaction | 0.00 | -0.0029,0.0028 | .98 | | -0.0003 | | -0.0036,0.0031 | .88 | | **-0.0115** | | **-0.0222,-0.0008** | **.036** |
| Self-realisation | -0.0134 | -0.0193,-0.0076 | **<.001** | | -0.0136 | | -0.0198,-0.0074 | **<.001** | | **-0.0647** | | **-0.0870,-0.0425** | **<.001** |
| Control-autonomy | -0.0081 | -0.0139,-0.0024 | **.006** | | -0.0095 | | -0.0163,-0.0027 | **.006** | | **-0.0298** | | **-0.0562,-0.0034** | **.027** |
| Additionally adjusted for covariates | | | | | | | |  |  | |  | |  |
| Positive affect | 0.002 | -0.0060,0.0100 | .62 | | 0.0005 | | -0.0083,0.0092 | .92 | | **-0.0323** | | **-0.0604,-0.0041** | **.025** |
| Life satisfaction | 0.0012 | -0.0017,0.0041 | .41 | | 0.0011 | | -0.0022,0.0044 | .50 | | -0.0059 | | -0.0165,0.0047 | .27 |
| Self-realisation | -0.0095 | -0.0154,-0.0036 | **.002** | | -0.0091 | | -0.0155,-0.0028 | **.005** | | **-0.0476** | | **-0.0700,-0.0252** | **<.001** |
| Control-autonomy | -0.0047 | -0.0106,0.0011 | .11 | | -0.0058 | | -0.0126,0.0011 | .098 | | -0.0154 | | -0.0409,0.0101 | .23 |

*WBC: white blood cell count; Unadjusted models automatically account for all factors that are time-constant in older age, including age, sex, ethnicity, education, socio-economic status, genetics, past history of mental illness, and past history of wellbeing. Mental ill health includes depression and other psychiatric conditions. Additional covariates include chronic conditions and smoking. Boldface indicates p<.05*
